# *Mycobacterium chelonae* hand infection following acupuncture: a case report and literature review

**DOI:** 10.3389/fmed.2024.1482236

**Published:** 2024-11-21

**Authors:** Sladjana Matic, Valerija Teodosic, Slavisa Zagorac

**Affiliations:** ^1^Faculty of Medicine, University of Belgrade, Belgrade, Serbia; ^2^Clinic for Orthopedic Surgery and Traumatology, University Clinical Center of Serbia, Belgrade, Serbia

**Keywords:** *Mycobacterium chelonae*, acupuncture, hand infection, surgical excision, non tubercolous mycobacteria

## Abstract

Hand infection caused by atypical mycobacteria is an uncommon condition. We present a case of hand infection caused by *Mycobacterium chelonae* in a patient who had undergone acupuncture. The clinical features, treatment, and outcome are described. Biopsy and cultures are essential for the diagnosis because *Mycobacterium chelonae* is a rare cause of human infection and is difficult to diagnose unless suspected. The patient was successfully treated through a combination of surgical excision, debridement, and antimicrobial therapy. We also reviewed the available literature to summarize the experience related to this infectious entity.

## Introduction

Acupuncture is a form of alternative medicine that treats patients by inserting thin needles into the body. This practice involves piercing the skin with solid, hair-thin needles that vary in length from 15 to 50 mm. The earliest written record of acupuncture dates back to approximately 200 BC (1). In the concept of traditional Chinese medicine, it is believed that acupuncture promotes general health, relieves pain, and prevents disease. There is general agreement that acupuncture is safe when administered by well-trained practitioners using sterile needles (2). However, it is an invasive procedure because the needles penetrate the skin and, therefore, is not without risk. The majority of reported adverse effects are minor, including hematoma (2.2%), minor hemorrhage (2.9%), and dizziness (1%) (2). Since 1970, 50 cases of bacterial infections and more than 80 cases of hepatitis B have been reported in the literature (3). Other risks include nerve injury, kidney damage, hemopericardium, and even brain damage (4).

Non-tuberculous (atypical) mycobacteria are ubiquitous organisms, commonly found in the environment (soil, water, and dust) and animal reservoirs (5).

Other organisms of the *Mycobacterium* genus that are present in the environment are classified as saprophytes and have been referred to as “atypical mycobacteria” by Pinner (6).

Runyon distinguished between rapidly growing and slowly growing non-tuberculous mycobacteria (NTM), further subdividing them according to their pigment-forming properties in culture. *Mycobacterium chelonae* (*M. chelonae*) is an atypical rapidly growing mycobacterium (RGM) belonging to Runyon group IV. *M. chelonae* was originally isolated from a turtle (7), and it is widely found in soil, freshwater, and dust particles.

*M. chelonae* infection in the hand is rare (8). Here, we present a case of *M. chelonae* infection in an immunocompetent patient following multiple acupuncture treatments. We reviewed the available literature to summarize the experience related to this infection.

## Case report

**Figure 1:**
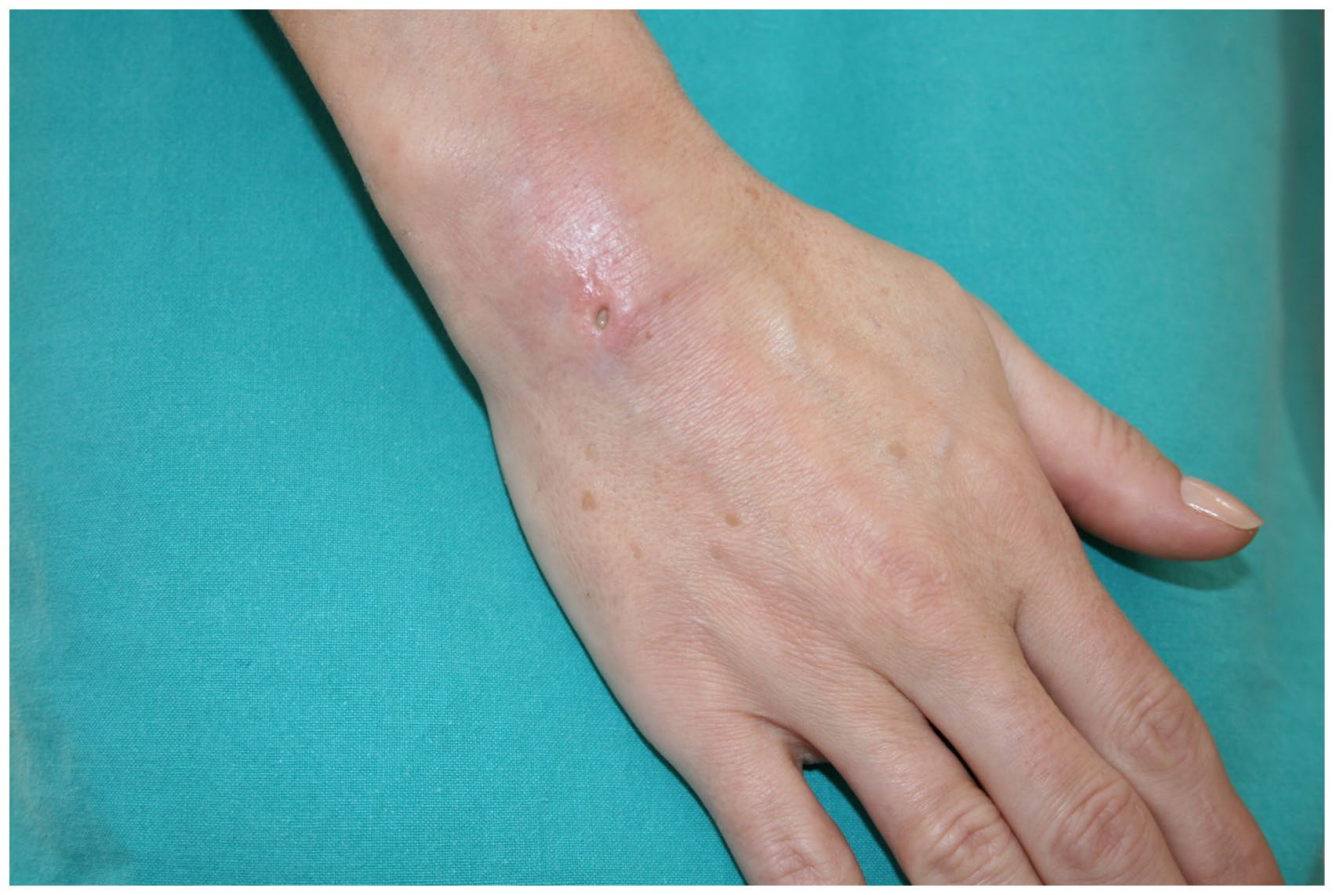
Punched-out ulceration on the patient’s right hand.

We present the case of a 45-year-old right-handed woman with a punched-out ulceration on her right hand (Figure 1). She presented to her general practitioner in February 2023 with a tender, red, suppurating subcutaneous nodule on the skin of the dorsum of her hand. 3 weeks before the examination, the patient underwent acupuncture treatment for chronic lumbar and shoulder pain. She was initially treated with amoxicillin (500 mg, three times a day, orally), followed by cefazolin (500 mg, twice a day, intravenously), but with no improvement. The routine cultures were negative, while mycobacterial cultures were not requested. The patient initially complained of a prickling sensation around the lesion, accompanied by itchiness. No systemic symptoms, such as fever or myalgia, were reported.

In May 2023, the patient was referred to a dermatologist due to moderate swelling and a tender erythematous nodule with purulent discharge on the dorsum of the right hand and wrist. The induration was located at the site of the acupuncture point that lies along the triple heater meridian (meridian X). Based on the clinical findings, the patient was treated with ciprofloxacin (500 mg, twice a day, orally) for 10 days. Despite completing the full course of this treatment, discharge from the wound continued. The hepatic and renal function tests were normal. The blood tests showed the following: erythrocyte sedimentation rate = 4 mm/h (reference range: 3–20 mm/h), C-reactive protein = 3 mg/L (reference range: 0.1–8 mg/L), and mean WBC = 3.8 × 10^9^/l (reference range: 3.4–9.7 × 10^9^/l). The rheumatoid factor, antinuclear antibodies, and serological tests for Chlamydia were negative. The Widal, Weil–Felix, and HIV A1 and A2 tests were also negative. The blood culture was sterile.

The hand X-rays showed swelling of the soft tissue on the dorsum of the hand, without any joint or bone erosions.

The patient’s chest X-ray was normal, and the Mantoux test was also negative. The samples from the wound swabs were inoculated onto appropriate nutrient media, including nutrient agar (blood agar, Endo agar, mannitol salt agar (Chapman agar), MacConkey agar, Sabouraud agar, crystal violet blood agar, and Pseudosel agar), as well as nutrient broth (brain heart infusion broth, thioglycollate broth, and Robertson’s Cooked Meat (RCM) medium). All cultures yielded no growth after an incubation period of 16–24 h at a temperature of 35–37°C.

The patient was afebrile and in good overall health. There was no indication of immunodeficiency. The laboratory tests revealed no fungal elements. The patient had no history of systemic immunosuppressive therapy.

The Ziehl–Neelsen stain for acid-fast bacilli was positive. The culture on Löwenstein–Jensen medium showed non-pigmented colonies on the fourth day of the inoculation, which were preliminarily identified as a rapidly growing non-tuberculous mycobacterium. The definitive identification of the pathogen as *M. chelonae* was based on targeting the mycobacterial *360-bp* region of the *rpoB* gene, which is relatively polymorphic, while it was amplified from the DNA by PCR-restriction fragment length polymorphism analysis (9, 10).

**Figure 2:**
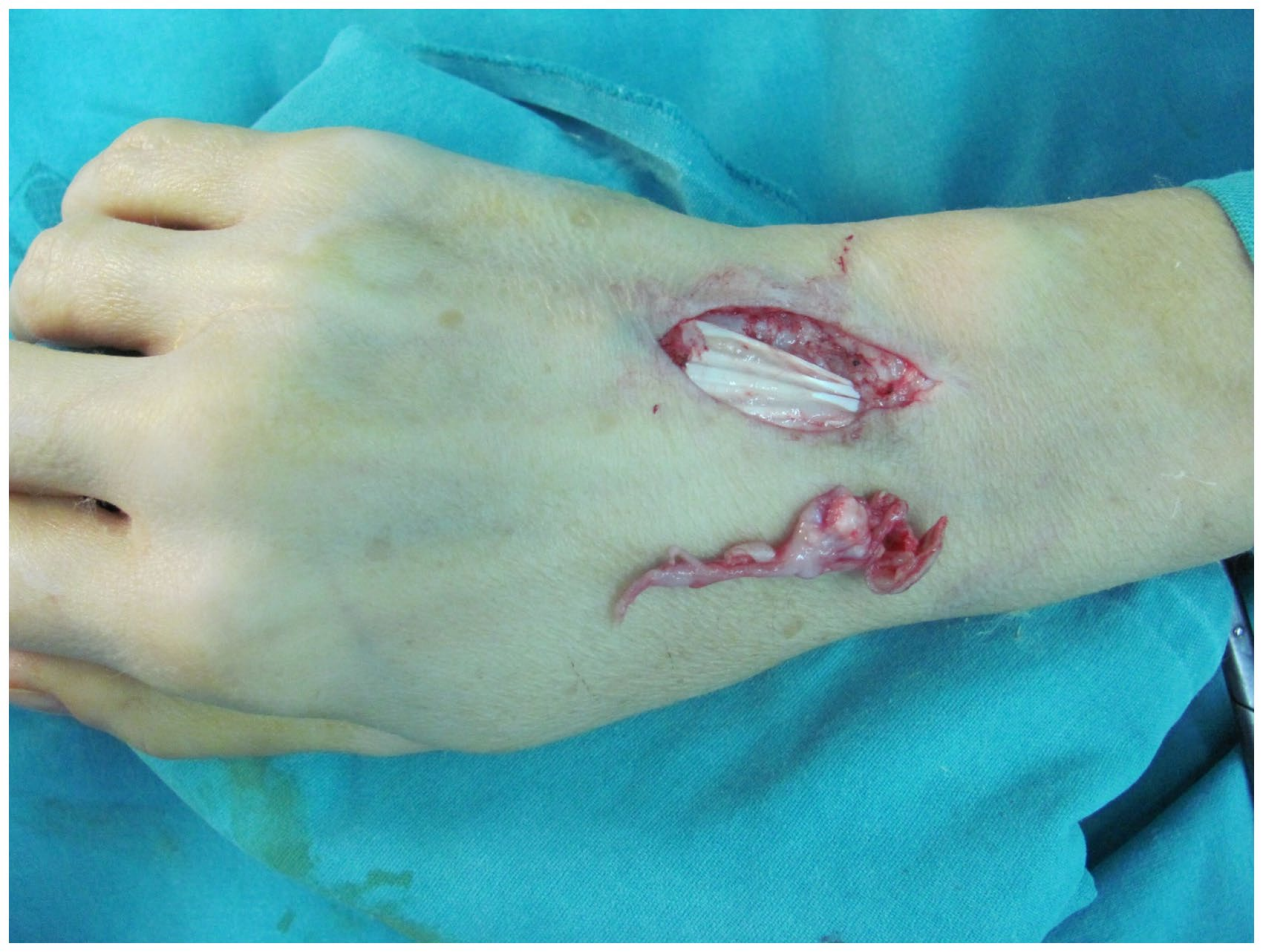
Intraoperative view showing excision of the lesion and tenosynovectomy of the extensor tendons.

Treatment with clarithromycin (500 mg twice a day, orally) and tobramycin (3 mg/kg/day, intravenously) was initiated for 2 weeks, and there was a positive clinical response to this treatment. The patient did not experience any gastrointestinal side effects. Clarithromycin monotherapy was continued for the remaining of her planned 6-month treatment course. The lesion was excised, and tenosynovectomy of the extensor tendons (Figure 2) was performed. The biopsy sample taken from the soft tissue was sent for microbiological and pathological analyses. Histologically, the biopsy specimens revealed signs of acute inflammation superimposed on chronic dermal and subcutaneous inflammatory lesions. Polymorphonuclear leukocyte infiltration was considerable and associated with signs of granulomatous inflammation. Foreign-body giant cells, Langerhans cells, and histiocytes were present in the biopsy sample.

**Figure 3:**
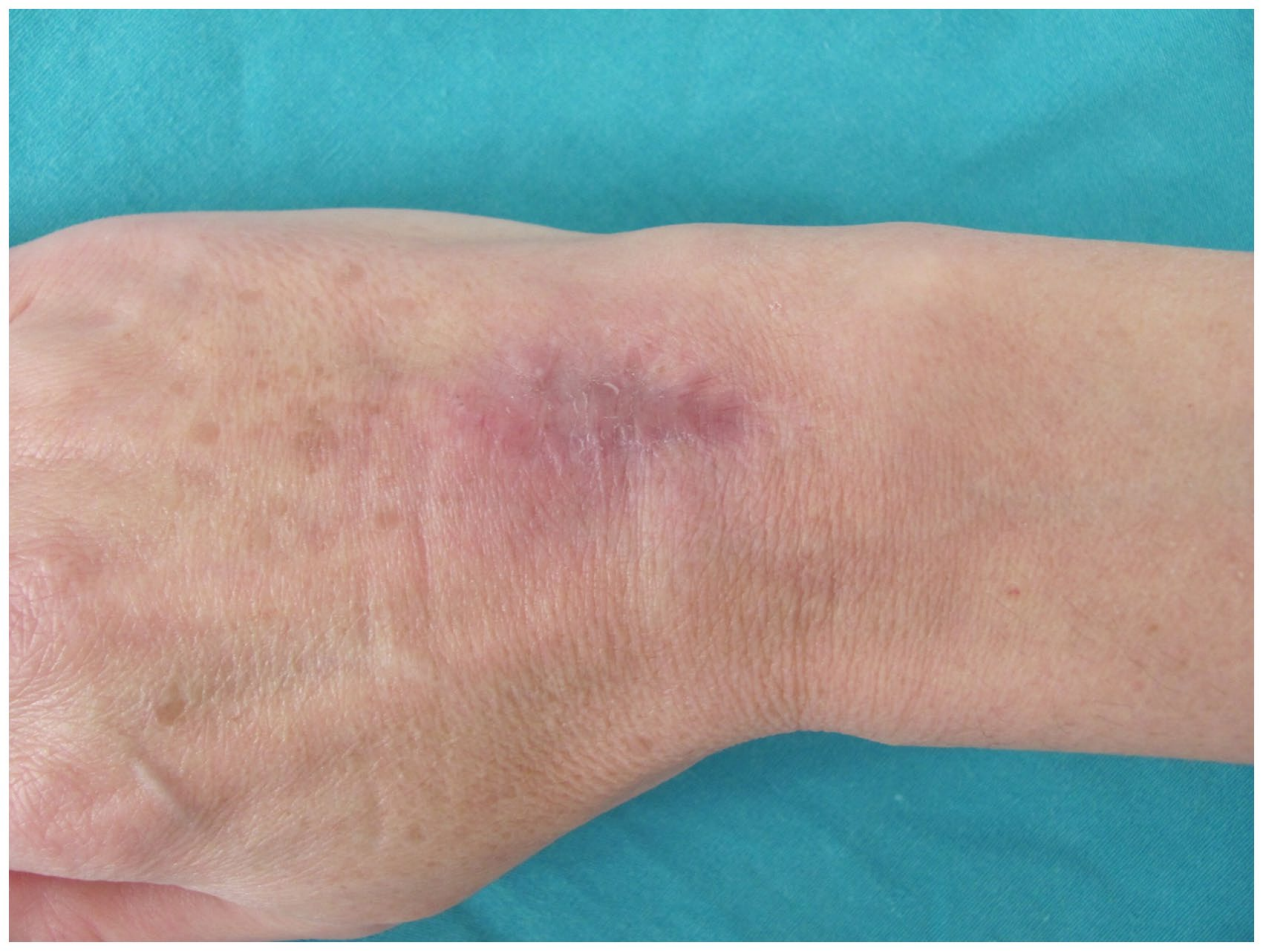
Wound completely healed without any further discharge from the site, with residual scarring and hyperpigmentation.

**Figure 4:**
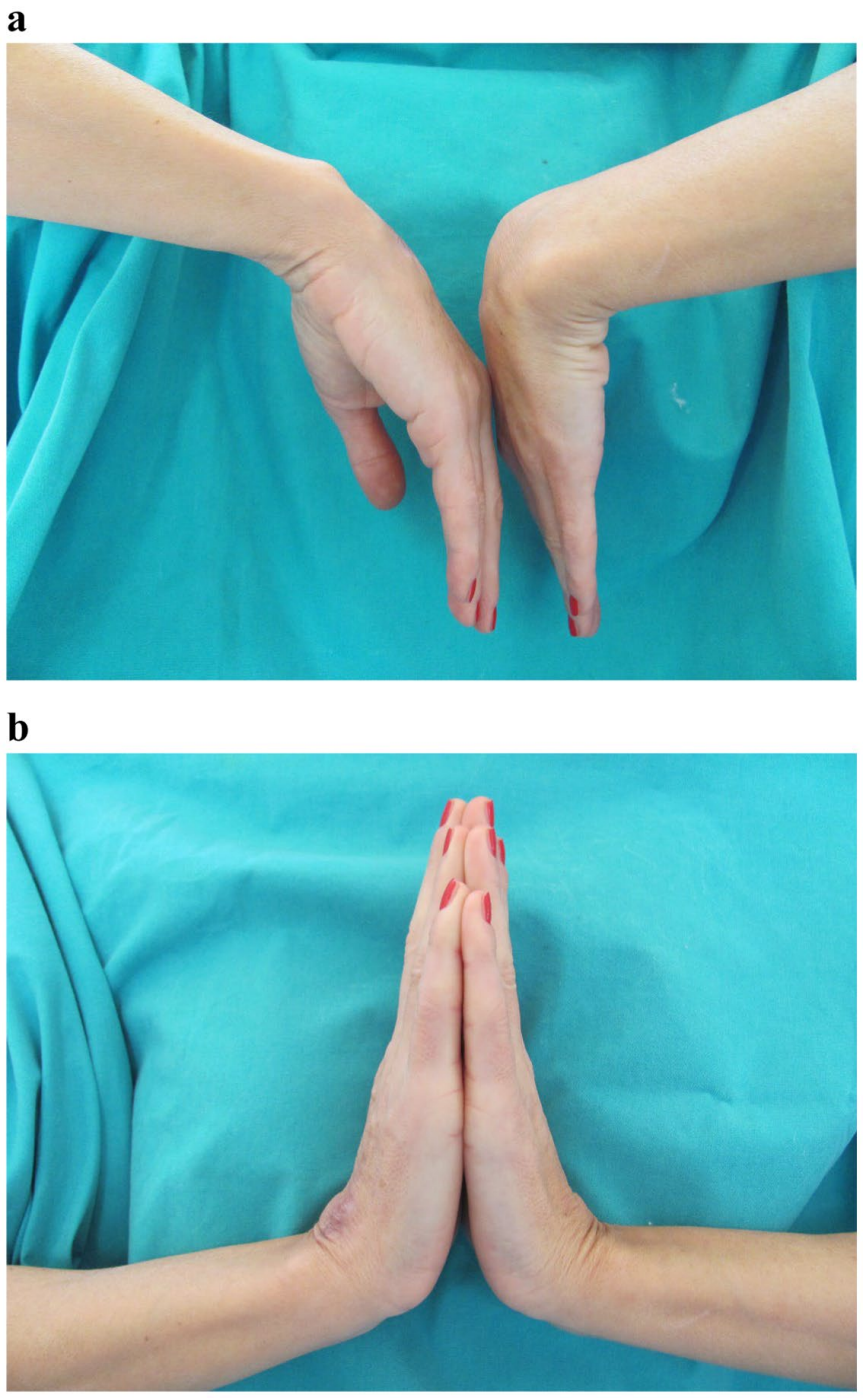
**(a)** Range of motion; palmar flexion. **(b)** Range of motion; dorsiflexion.

After 4 months of the treatment, the edema resolved, discharge from the wound ceased, and the nodules disappeared. After 2 months, the treatment was discontinued. The incision site healed with no signs of infection. To prevent adhesions, occupational hand therapy consisting of a wide range of motion exercises was carried out. The final assessment was condcuted after 6 months. There were no signs of infection. The wound completely healed without any further discharge from the wound site. The patient developed residual scarring and hyperpigmentation (Figure 3). The grip strength was measured using the Jamar Hydraulic Hand Dynamometer, with the elbow in 90° flexion and the wrist in a neutral position. The measurement showed that the patient regained 90% of the grip strength of the contralateral hand. She also regained a satisfactory range of motion, with almost full dorsiflexion and palmar flexion, reaching 75% of the contralateral hand (Figure 4). The Disabilities of the Arm, Shoulder, and Hand (DASH) score was 1.7.

## Discussion

Rapidly growing mycobacteria (RGM) are commonly found in soil, dust, and water. RGM are known to cause respiratory infections, lymphadenitis, localized cutaneous infections, chronic granulomatous infections of the bursae, joints, tendon sheaths, and bones, and, rarely, disseminated systemic illness (11). Depending on the host’s immune status, cutaneous disease caused by *M. chelonae* follows two different patterns (12). In an immunocompetent host, a traumatic injury, injection, or surgical wound is followed by the development of localized cellulitis or abscess formation. Disseminated disease is usually observed only in immunocompromised hosts, such as those with immunosuppression, AIDS, or those who use corticosteroids.

Cases or outbreaks of *M. chelonae* infection have been reported following liposuction (13), intramuscular injection (14), vaccination (15), laparoscopy (16, 17), mesotherapy (18, 19), catheter infections (12), breast augmentation (20), silicon arthroplasty of the metacarpophalangeal joints (21), at the site of tattoos (22), open fractures (23), and carpal tunnel release procedures (24). A history of trauma preceded the appearance of lesions in 44% of patients (25).

Terry et al. reported an *M. chelonae* ulcerous lesion on the leg of a patient with a history of diabetes mellitus, following a scratch from a cat (26). Other authors have reported mycobacterium tenosynovitis of the hand and wrist without a recognized penetrating injury (8, 24, 27). Chung et al. reported bilateral cutaneous *M. chelonae* infection on both lower legs with no history of trauma, while Sari-Kouzel et al. reported pustules on a finger associated with Raynaud’s phenomenon (28, 29). Uslu et al. reported five cases of cutaneous and/or soft tissue infections due to *M. chelonae*, where two of the five patients were receiving immunosuppressive treatment (30).

We found several reports of cases and outbreaks of skin and soft tissue infections caused by *M. abscessus* following acupuncture in the literature (31–35), as well as two articles reporting on *M. chelonae* infection following acupuncture treatment: induration of the leg in a 79-year-old woman and disseminated subcutaneous nodules on the skin of the abdomen of a 58-year-old woman (36, 37). To the best of our knowledge, this is the first reported case of *M. chelonae* infection in the hand following acupuncture treatment in the literature. The exact source of the infection was not determined. However, there are several possible explanations. First, the acupuncture needle itself could have been the source of the infection due to improper sterilization. A second possibility is skin contamination before or after acupuncture by an object in the environment, such as disposable needles, towels, alcohol bottles, or water from the water heater. This was demonstrated in the case presented by Chroneou et al., where a positive culture of *M. chelonae* was found in an automated bronchoscope washer and the water supply (38).

In the majority of postinjection abscesses caused by *M. chelonae* and *M. abscessus*, the contaminated source was not identified. However, in general, non-sterile water used to dissolve different substances has been suggested as the source of the pathogen (37, 39). In an effort to identify the source of *M. abscessus* infection following acupuncture treatment, Koh et al. investigated an outbreak among patients who had visited an oriental medical clinic (34). Fifty environmental samples were collected from the clinic for mycobacterium culture inoculation. These samples were taken from all devices and materials that could have come into contact with the patients’ skin before, during, and after the acupuncture sessions. However, none of the cited studies have elucidated the source of the infection or the mode of transmission. In two articles, the authors reported that the source of *M. chelonae* infection after mesotherapy was the tap water system in the treating physician’s office, which was used to rinse the injector (18, 19).

Diagnosing cutaneous infections caused by rapidly growing mycobacteria is difficult for several reasons. First, the clinical symptoms of these infections are often non-specific, such as abscesses or subcutaneous nodules, without systemic features (19). The morphology of skin lesions is highly heterogeneous. Some lesions remain in the form of papules, whereas other lesions progress to nodules, abscesses, ulcers, and even confluent plaques filled with pus (25, 31). In some cases, patients were found to delay seeking medical advice because the symptoms were relatively mild (36). Second, the incubation period can vary. In our case, the onset of symptoms occurred 3 weeks after the acupuncture treatment. The literature reports a wide range of incubation periods: a median incubation period of 9 days or 9.5 weeks (range: 1–29 weeks) after mesotherapy (18, 19) and approximately 3–5 months after multiple acupuncture treatments (33, 37, 40). In some cases, patients failed to associate the acupuncture procedures with cutaneous lesions because of the long incubation period (36). The average delay between the onset of symptoms and the correct diagnosis has been found to be 1 year (41). A clinical suspicion of cutaneous non-tuberculous infections, such as *M. chelonae* infection, is essential for diagnosis. The symptoms typically include prolonged or failed wound healing and a lack of response to broad-spectrum antibiotics in cases of suspected bacterial infection.

These pathogens are difficult to treat once the infection is diagnosed. Mortality typically occurs only in cases of extensive pulmonary or disseminated disease. No physical examination findings are pathognomonic for *M. chelonae* infection. In the differential diagnosis, conditions such as wound infection, actinomycosis, blastomycosis, sporotrichosis, histoplasmosis, fungal infection, and other mycobacterial infections must be considered (42–44).

After establishing the diagnosis, susceptibility testing should be performed. *M. chelonae* is usually susceptible to tobramycin, clarithromycin, and linezolid (19). Some studies have reported a 100% susceptibility to ciprofloxacin and clarithromycin (25).

The type and duration of antimicrobial therapy for *M. chelonae* infection vary considerably in the literature. Although numerous reports have documented cases of successful monotherapy (e.g., clarithromycin, 1 g a day or 500 mg twice a day, orally) (21, 22, 26, 28, 37), antibiotic therapy with two or three drugs is preferable in most patients (14, 29, 36). The duration of antibiotic treatment may vary from as little as 5 weeks (29) to as long as 3 months (28, 37), 5 months (17), or even 9 months (8). The majority of authors have recommended 6 months (19, 21, 23, 36, 39, 45) or a total of 12 months of treatment (46).

Another important consideration is whether surgical procedures are necessary in such cases. Some authors consider that a prompt diagnosis of atypical mycobacteria infections, along with appropriate antimicrobial treatment, may help avoid the need for surgical debridement (8). In two cases of *M. chelonae* infection following acupuncture treatment, antibiotics alone were sufficient to completely clear the lesions (36, 37). In other articles, we found that the standard guidelines typically include both antibiotic treatment and surgical management. This management involves debridement of affected skin and tissues, irrigation, removal of existing implants, and drainage (21, 26, 28, 29, 39, 45, 47, 48).

*M. chelonae* is a rapidly growing mycobacterium responsible for a variety of generally chronic infections (44, 49, 50). The infections sometimes develop after surgery or trauma and affect the skin and soft tissues. Localized infections without a history of trauma are uncommon.

Sterilization and the use of disinfectants play a crucial role in the proper control of NTM, especially in healthcare settings (51). Glutaraldehyde, peracetic acid, povidone-iodine, alcohol, and chlorine are still used for the sterilization of medical instruments, and common methods of water disinfection include chlorine, monochloramine, and ozone. NTM are resistant to chlorine and other disinfectants used in drinking water systems; however, most disinfectants have some sterilizing effects against NTM (52, 53). Lee et al. (53) showed that glutaraldehyde has the greatest sterilizing effect on non-tuberculous mycobacteria, while only alkyldiaminoethylglycine hydrochloride has a reduced effect on NTM. Glutaraldehyde-resistant strains of *M. chelonae* have been isolated from environmental samples. An alternative disinfectant, such as glucoprotamin, often shows good efficiency against the majority of the different NTM isolates (e.g., *M. smegmatis*, *M. avium*, *M. kansasii*, *M. terrae*, and *M. xenopi*), except for *M. chelonae* (51, 54).

Biopsy and cultures are essential for the diagnosis. The optimal treatment for cutaneous infections caused by *M. chelonae* is yet to be established. Effective treatment of these infections is therefore challenging, and the identification of antimicrobial susceptibility is essential. Clarithromycin has been reported as the most effective drug for treating *M. chelonae* infection (55, 56). Awareness of the spectrum of clinical presentations of this infection can help prevent delays in diagnosis and facilitate the timely application of appropriate antibiotic and surgical therapies.

## Conclusion

Non-tuberculous mycobacteria should be recognized as a preventable cause of acupuncture-associated infections. Atypical mycobacterial infections should be considered when skin lesions develop following any procedure that involves the subcutaneous tissue. Proper handling of equipment, appropriate skin preparation, and strict adherence to sterilization procedures can help prevent future cases and outbreaks of *M. chelonae* infection. Efforts should focus on educating acupuncture practitioners about hygienic practices based on established guidelines, especially those related to invasive procedures in non-hospital settings. Moreover, future epidemiological studies are needed to identify the exact source of the infection.

## Data Availability

The raw data supporting the conclusions of this article will be made available by the authors, without undue reservation.
